# Molecular and Genetic Characterization of Colicinogenic *Escherichia coli* Strains Active against Shiga Toxin-Producing *Escherichia coli* O157:H7

**DOI:** 10.3390/foods12142676

**Published:** 2023-07-11

**Authors:** Mauro D. García, María J. Ruiz, Luis M. Medina, Roberto Vidal, Nora L. Padola, Analía I. Etcheverria

**Affiliations:** 1 Laboratorio de Inmunoquímica y Biotecnología, Centro de Investigación Veterinaria de Tandil (CIVETAN), CONICET, CICPBA, Facultad de Ciencias Veterinarias, UNICEN-Campus Universitario, Tandil B7000, Argentinajruiz@vet.unicen.edu.ar (M.J.R.);; 2 Food Science and Technology Department, Faculty of Veterinary Medicine, Universidad de Cordoba, 14071 Córdoba, Spain; 3 Instituto de Ciencias biomédicas, Facultad de Medicina, Universidad de Chile, Santiago 8380453, Chile

**Keywords:** STEC, *E. coli* O157:H7, colicin, genome

## Abstract

The objective of this work was to molecularly and genotypically characterize and test the inhibitory activity of six colicinogenic *Escherichia coli* strains (ColEc) and their partially purified colicins against STEC O157:H7 isolated from clinical human cases. Inhibition tests demonstrated the activity of these strains and their colicins against STEC O157:H7. By PCR it was possible to detect colicins Ia, E7, and B and microcins M, H47, C7, and J25. By genome sequencing of two selected ColEc strains, it was possible to identify additional colicins such as E1 and Ib. No genes coding for *stx1* and *stx2* were detected after analyzing the genome sequence. The inhibitory activity of ColEc against STEC O157:H7 used as an indicator showed that colicins are potent growth inhibitors of *E. coli* O157:H7, being a potential alternative to reduce the presence of pathogens of public health relevance.

## 1. Introduction

*Escherichia coli* is a Gram-negative bacterium, part of the normal microbiota of the intestinal tract of warm-blooded animals, including humans. Most of them are nonpathogenic, but some can cause diseases such as watery diarrhea (WD), hemorrhagic colitis (HC), and hemolytic uremic syndrome (HUS). This disease, particularly severe in young children, is caused by Shiga toxin-producing *Escherichia coli* strains (STEC) that synthesize potent toxins and other virulence and adherence factors that contribute to their pathogenicity [1]. The main serotype associated with HUS worldwide is *E. coli* O157:H7 [2]. However, some non-O157 serotypes are frequently associated with HC and HUS in several countries. Argentina has the greatest worldwide prevalence, with an average incidence for HUS of 16/100,000 children younger than 5 years, without considering the sub-recording of the disease [3]. A report from Argentina in response to an FAO/WHO request confirmed that the etiologic agent most associated with HUS is STEC, with O157:H7 being the most frequently associated serotype, although there are more than 100 serotypes that have similar pathogenic potential [4]. STEC infection is associated with consumption and contact with contaminated foods and water, person-to-person transmission, and direct contact with infected animals, their feces, or their environment [5,6].

Bacterial communities are forced to compete with each other for nutrients and energy sources. They achieve this by producing proteins that inhibit the growth of phylogenetically related (reduced spectrum) or not-related (broad spectrum) strains. Bacteriocins are an important type of antimicrobial ribosomally synthesized peptides (in contrast with antibiotics, which are secondary metabolism products) and are present in all Eubacteria and Archaea genera [7,8].

In addition to their use as biopreservatives antagonistic to FBD (food-borne diseases) pathogens, bacteriocins have potential novel applications in animal and human health. Therefore, the search for producer strains and the functional characterization of these molecules is of paramount importance. Currently, the only bacteriocin approved worldwide for use as a biopreservative agent is nisin, synthesized by *Lactococcus lactis*, which has proven to be effective against multiple important pathogens, including *Listeria monocytogenes*, *Clostridium botulinum*, *E. coli*, and *Salmonella* spp. The use of nisin is not restricted only to the application in food but is marketed in disinfectant preparations for topical and intramammary application, applied to dairy cattle with mastitis due to *Staphylococcus aureus*, *Streptococcus uberis*, and *Streptococcus agalactiae* [9]. Nisin works by forming pores in the cell membrane, causing the leaching of important cellular compounds, including K+ ions, amino acids, and ATP, ultimately leading to cell death [10]. One of the main applications of bacteriocins in human health is the treatment of multi-resistant bacteria responsible for hospital-acquired infections [11].

Although the appearance of resistance or tolerance to bacteriocins is a factor to be considered in therapeutic applications, an alternative would be the use of combinations of bacteriocins from different origins or bacteriocins modified in their peptide sequence to evade the resistance acquired by target cells.

Colicins are the most studied bacteriocins produced by Gram-negative bacteria, with 25 different types being identified [12,13].

In *E. coli*, bacteriocins are classified based on their molecular weight as colicins (25–80 KDa) and microcins (smaller than 10 KDa). Colicins and microcins have a relatively narrow spectrum of activity, although, in general, microcins have wider antibacterial activity compared to colicins [14]. The colicin operons are encoded on the bacterial chromosome or on colicinogenic plasmids (pCol) that harbor, in addition to the colicin gene itself, all the genes necessary for their transport and immunity [13]. Among these classes of bacteriocin are colicins E1–E9, Ia, Ib, and S4. Microcins are frequently coded in the bacterial chromosome, and examples of these are microcins H47, M, or J25, among others. The action mechanism consists of the formation of pores in the target cell wall, the inhibition of peptidoglycan synthesis, and the inhibition of DNA replication or RNA transcription [15]. Colicin’s expression varies according to the bacterial growth phase, which in some cases occurs during the exponential phase [16,17], while in others, it occurs in the stationary phase [18,19].

Most of the bacteria considered probiotics belong to the *Lactobacillus*, *Enterobacter*, and *Bifidobacterium* genera [20,21]. However, the use of nonpathogenic strains of *E. coli* has been considered to achieve the reduction of infections caused by pathogenic bacteria in both animals and humans [22]. The use of *E. coli* as a probiotic is mainly accepted in Germany and other Eastern European countries. Wassenaar et al. (2016) [23] pointed out that *E. coli* is chosen as a probiotic in line with its presumed ubiquitous presence in the gut. An example is the *E. coli* Nissle 1917 strain, widely used in probiotic preparations. Colicins and colicin-like molecules derived from Gram-negative bacteria are easily and well-expressed in plants and fully functional, being up to 10^6^ times more potent than antibiotics on a molar basis [24]. A promising alternative to circumvent the existing sanitary concerns regarding the use of *E. coli* under viable conditions is the use of colicins produced against pathogenic bacteria [25,26]. Numerous groups are constantly searching for new strains and new types of colicins [27,28], trying to express known colicins in different combinations [29] or new organisms, such as plants, to achieve higher yields and purity of the peptide extract [30].

The objective of this work was to molecularly and genotypically characterize and test the inhibitory activity of colicinogenic *E. coli* strains (ColEc) and their partially purified colicins against STEC O157:H7 isolated from clinical human cases.

## 2. Materials and Methods

### 2.1. Origin and Identification of E. coli and Indicator Strains

In this study, six strains isolated in 2004 from the bovine gastrointestinal tract were used and identified as *E. coli* 4.8, *E. coli* 8.2, *E. coli* 13.7, *E. coli* 24.7, *E. coli* 27.4, and *E. coli* 27.12. Colicinogenic *E. coli* strains were then identified as ColEc. These strains previously showed the absence of the different virulence factors: Shiga toxin (*stx1* and *stx2*), intimin (*eae*), hemolysin (*hlyA*), thermo-stable toxin (*stIa*), and thermo-labile toxin (*ltI*) by polymerase chain reaction (PCR) genotypic analysis [31].

The absence of virulence genes was confirmed by a complementary study of the genetic profile using the PCR technique. This study included the associated genes, plus adhesion-associated genes such as intimin for *E. coli* O157 (*eaeO157)*, hemolysin (*ehxA*), *efa*/*iha*, antigen 43 (*AGN43*), and intracellular domain associated protein-1 (*AIDA1*) factors.

#### 2.1.1. Genetic Profiles by Random Amplification of Polymorphic DNA (RAPD)

The determination of the genetic profiles of the selected bacteria was carried out using the random amplification of polymorphic DNA (RAPD) technique. The technique was performed based on the methodology developed by Birch et al. [32] with modifications. An aliquot of the tubes with the bacteria in stock (frozen at −80 °C) was seeded in 10 mL of Luria Bertani broth (LB, Britania, Argentina) and incubated at 37 °C under agitation for 18 h. Then, the OD_600_ of a 1/10 dilution of each culture was determined. For an OD value of 0.5, 500 μL of the culture was taken and centrifuged for 2 min at 12,000× *g*. The pellet obtained was resuspended in 500 μL of bidistilled water. The suspension obtained was boiled for 10 min, placed on ice, and centrifuged for 3 min at 13,000× *g*. The supernatant was extracted and frozen at −80 °C for later use. 

The amplification was performed using the primer [32] M13: 5′GAGGGTGGCGGTTCT 3′. The PCR reaction cocktail was made in a final volume of 50 µL containing 20 mM (NH_4_)_2_SO_4_; 75 mM Tris-HCl pH 9.0; 0.1% (*w*/*v*) Tween 20; 2.5 mM MgCl_2_; 0.2 mM of each dNTP; 1 µM of the “primer”; 2.5 U of Taq DNA polymerase (Highway, Argentina); and 5 μL of the sample. The PCR reaction was performed in a thermocycler (Techne Genius, Thermo Fisher, Waltham, MA, USA) under the following conditions: initial temperature of 94 °C for 5 min, 40 cycles of 94 °C for 1 min, 50 °C for 1 min, and 72 °C for 1 min. The amplification products were analyzed by horizontal electrophoresis on a 1.8% agarose gel in the presence of ethidium bromide.

#### 2.1.2. Serotyping

Serotyping O and H antigens were determined by means of a microagglutination technique in plates and tubes described by Guinée et al. [33] modified by Blanco et al. [34,35] using all available O (O1–O175) antisera plus six putative new O antigens (OX176 through OX181) [36] and H (H1–H56) antisera [37]. Non-specific agglutinins were removed by adsorption with the corresponding cross-reacting antigens. O antisera were produced in the Laboratory Reference of *E. coli* (LREC) (Lugo, Spain), whereas the H antisera were obtained from the Statens Serum Institut (Copenhagen, Denmark). 

#### 2.1.3. Origin of Indicator Strains

STEC O157:H7 was used as an indicator for the inhibitory activity of ColEc. STEC O157:H7 (*n* = 80) strains isolated between 1996 and 2013 from Argentinean and Chilean patients in clinical case studies of HUS, HC, and WD were used (unpublished data, Table 1). In addition, a STEC strain (identified as STEC 166), isolated from grazing cattle, was used [31].

### 2.2. Detection of Colicin Genes

ColEc were cultured on LB broth (Britania, Argentina) (overnight -ON- at 37 °C) with continuous shaking. DNA extraction was carried out by boiling 10 μL of this culture in 500 μL of sterile bidistilled water for 10 min.

Colicin genes were identified in colicinogenic strains by PCR monoplex using the primers listed in Table 2 [36,37]. In a final reaction volume of 25 μL, 2.5 μL DNA extract, 4 mM MgCl_2_, 0.5 units of Platinum Taq (Invitrogen), 1× reaction buffer (67 mM Tris/HCl -pH 8,8-, 16.6 mM (NH_4_)_2_SO_4_, 0.45% Triton X-100), 0.2 mg of gelatin, 0.2 mM dNTP, and 3 ng of each primer were used. The amplification cycle consisted of an initial step of 94 °C for 2 min, 30 cycles of 94 °C for 30 s, 55 °C for 30 s, 72 °C for 30 s, and a final step at 72 °C for 3 min. Samples were electrophoresed on 2% agarose gels stained with ethidium bromide and viewed under ultraviolet light.

### 2.3. Determination of Inhibitory Activity of ColEc against STEC Strains

Assessment of the inhibitory activity of the ColEc strains was performed by puncture inhibition assay. First, the LB plates were punctured with each of the ColEc strains and incubated overnight at 37 °C. The colonies obtained were exposed to chloroform vapors for 1 h and then allowed to aerate for 30 min. Secondly, a soft agar containing 10^6^ CFU/mL of each pathogenic strain supplied for this study was poured on them. The same procedure was carried out to rule out a possible inhibitory effect of the pathogenic strains over colicinogenic strains in the study. The presence of inhibition was considered when a translucent zone around the colony with the absence of growth of at least 1 mm was observed. The inhibitory tests were carried out in triplicate, and inhibition of the indicator strains was observed in all cases.

#### Determination of Inhibitory Activity of Colicins against STEC 166

Two ColEc strains (24.7 and 27.4) that inhibited the highest number of STEC strains from HUS and HC used were selected to characterize their colicin inhibitory activity. Strain 27.4, although it was not the one that obtained the highest percentage, was selected due to the optimal concentration and purity of the DNA obtained during the extraction process. The colicins were partially purified by obtaining a cell-free supernatant (CFS). The overnight cultures were centrifuged at 13,000× *g* and filtered with a 0.22 µm pore size syringe filter. The CFS was placed in a 96-well microplate at different concentrations, inoculated with 10^3^ CFU/mL of STEC 166, and incubated at 37 °C. The average of the antimicrobial activity was determined by measuring optical density (OD_495nm_) each hour and by plate counting at 0, 2, 4, 6, and 8 h. These experiments were performed in triplicate, in three independent trials, and analyzed with INFOSTAT. They were expressed as the mean minus the standard deviation.

### 2.4. Differentiation Assay of Growth Inhibition by Phage or Colicin

With each ColEc strain, an inhibition assay was performed as described above. A small portion was then removed from each zone of growth inhibition and resuspended in 1 mL of LB broth containing two drops of chloroform. It was vortexed and left to stand for 5 min and 100 uL of this suspension was added to 3 mL of 0.4% LB agar containing 10^6^ CFU/mL of the indicator strain STEC 166. The agar was poured onto a plate and incubated at 37 °C for 18 h. The presence of lysis plaques in each of the samples would show that the inhibition occurs due to the presence of bacteriophages. On the other hand, if confluent growth of the indicator strain is observed on the LB agar plates without the appearance of lysis plaques, it can be confirmed that the inhibition is produced by bacteriocins. 

### 2.5. Stability of CFS to Physic-Chemical Treatments

CFS of the sequenced ColEC strains were subjected to several treatments to assess the stability of the potential antimicrobial substance contained in it. Those treatments consisted in:

I. Temperature: The CFS extracts were heated in a thermal bath at 60 °C, 80 °C, and 100 °C; they were frozen at −20 and kept at 4 °C [38].
II. pH changes: The pH of the CFS extracts were adjusted to approximately 4.5, 7.0, and 9.0 using HCl (*Biopack* 30%) and NaOH (*Biopack* 10N) (Larsen et al., 1993).
III. Stability to protease: CFS extracts were incubated with Proteinase K (Sigma-Aldricht 20 ng/µL) at 37 °C for 15–60 min.

After each treatment, the CFS extracts’ activity was evaluated with the inhibition assay explained in point 2.4.

### 2.6. Virulence Genes Detection 

The genome sequences previously obtained were analyzed with the *Virulence Finder* service provided by the Center for Genomic Epidemiology (www.cge.food.dtu.dk/services/VirulenceFinder, accessed on 27 February 2023), selecting a 90% threshold ID. After loading the assemblies, the graphical output of the program allowed us to detect the presence or absence of Shiga toxin genes [39].

### 2.7. Strains Sequencing: Bacteriocin Mining

The DNA of the selected colicinogenic *E. coli* strains was extracted with the Wizard^®^ Genomic DNA Purification Kit (Promega, Fitchburg, WI, USA), its quality and concentration were assessed with NanoDrop spectrophotometer (Thermo Fisher, USA) and sent to MicrobesNG (Birmingham, UK) using the standard whole genome sequencing with 30× coverage service. 

The procedure run by the provider was as follows: genomic DNA was organized into libraries using the Nextera XT prep protocol. Then, they were sequenced through the Illumina Hi-Seq platform with 2 × 250 bp paired-end reads. Reads were trimmed using Trimmomatic (V 0.40), and the quality was assessed with the Samtools (V 1.4), Bedtools (V 2.29.2), and Bwa-mem (V 0.7.17) software. De novo assembly was carried out using SPAdes (V 3.15.4). Annotation was made by the RAST server. Assemblies’ metrics were calculated using QUAST (V 4.0). Next, the genera and family of each strain’s read map was calculated using the KRAKEN (V 2.0) software. Finally, an automated annotation was performed using PROKKA (V 1.14). 

Genomes sequences assemblies were further analyzed for bacteriocin genes. BAGEL4 is a platform developed for the identification of bacteriocins and other ribosomally synthesized and post-translationally modified peptides (RiPPs) [40]. FASTA format sequences were uploaded onto the input page, followed by a graphical output showing the Areas of Interest (AOI) where each potential bacteriocin, as well as the immunity and modification genes related to its production, are identified on a color scheme. To re-confirm their identity, a Blast-p analysis was performed on the NCBI.

## 3. Results

### 3.1. Genetic Profile and Serotype Determination

The results of the PCR genotyping test for the different virulence and adherence factors showed that all the selected bacteria were negative.

**Figure 1:**
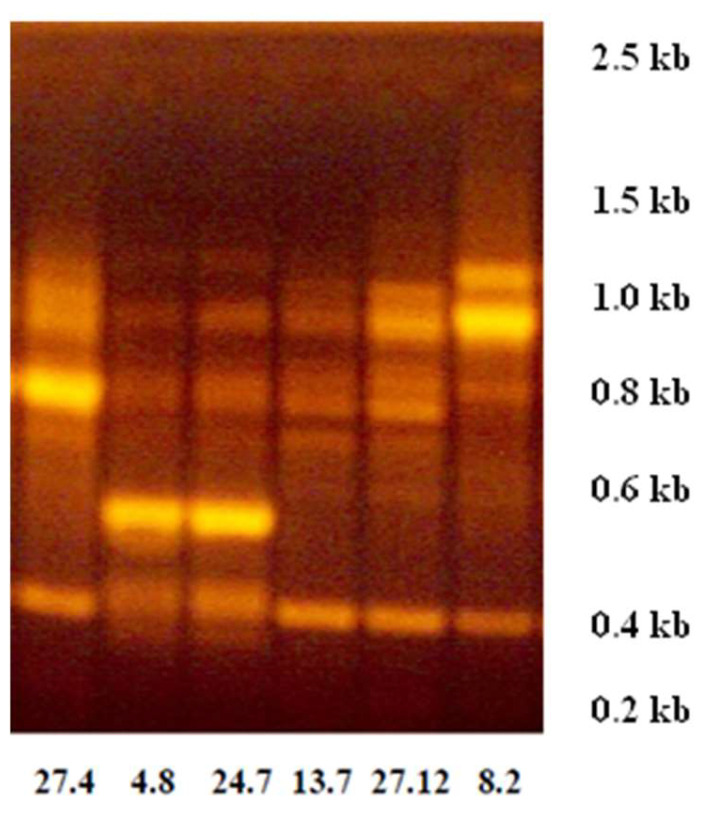
RAPD-PCR gel electrophoresis technique used to differentiate genetic profiles between selected ColEc strains. The numbers on the horizontal axis indicate the strain ID. The numbers on the vertical axis indicate the molecular size markers in kilobases (kb).

Using the RAPD technique, it was possible to differentiate four genetic profiles among the six ColEc strains (Figure 1). Strain selection was made based on the best ability to produce growth inhibition zones of the indicator strain in terms of halo size and clarity, established in a previous study [31]. Strains 4.8 and 24.7 shared one genetic profile, strains 13.7 and 27.12 shared another profile, while strains 8.2 and 24.7 showed a profile that was different from each other and different from the profiles of the other strains.

With the antisera used, it was possible to identify the *E. coli* 4.8 strain as ONT, the *E. coli* 8.2 strain as O77, the *E. coli* 13.7 strain as O174: H8, the *E. coli* 24.7 ONT: H7 strain, the *E. coli* 27.4 as O26 H-, and *E. coli* strain 27.12 as O141.

### 3.2. PCR Screening

By PCR assays, it was confirmed that the colicinogenic strains under study encode the following colicins: *E. coli* 4.8: colicins B, microcin M, and microcin H47; *E. coli* 8.2: colicin Ia and microcin C7; *E. coli* 13.7 colicins Ia, E7, B, and microcin C7 and microcin J25; *E. coli* 24.7 colicin B, microcin M, and H47; *E. coli* 27.4; colicin Ia, E7, B, microcin C7, and J25 (Table 3). With the primers used, it was not possible to identify colicins in strain *E. coli* 27.12, despite having inhibitory activity, as could be demonstrated in previous studies [36]. The correspondence of the strain name of the cited study with the present study is as follows: Strain 1: 4.8, Strain 2: 8.2, Strain 3: 10.10, Strain 4: 13.7, Strain 5: 24.7, Strain 6: 27.4, and Strain 7: 27.12. Particularly in the present study, strain 10.10 was discarded.

### 3.3. Inhibition Assays

The results of the puncture inhibition test using ColEc strains against STEC from HC and HUS are shown in Table 3. All colicinogenic strains were found to inhibit more than 90% of the pathogenic strains. The Petri dish puncture inhibition assay showed a zone of inhibition against most pathogenic strains. The positive reading was considered when three replicates showed halos greater than 1 mm.

The ColEc strains selected because of these results to characterize colicin inhibitory activity were ColEc 24.7 and ColEc 27.4 (Colicin produced by ColEc 27.12 could not be identified using the primers of Table 2, and no further tested according to Section 2.5).

**Figure 2:**
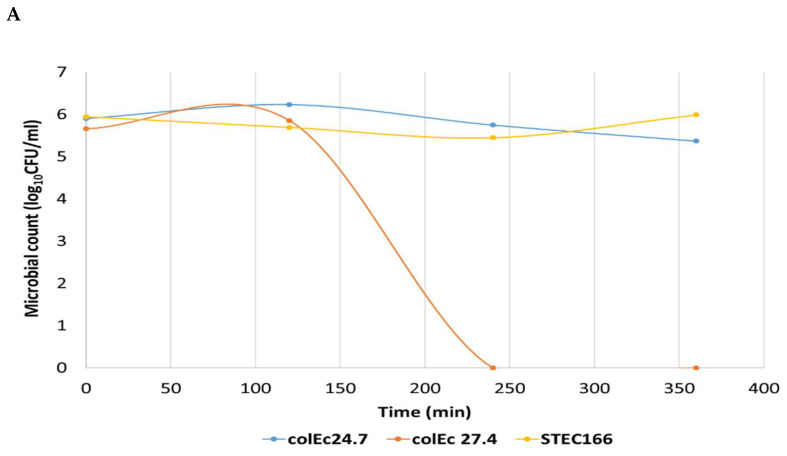

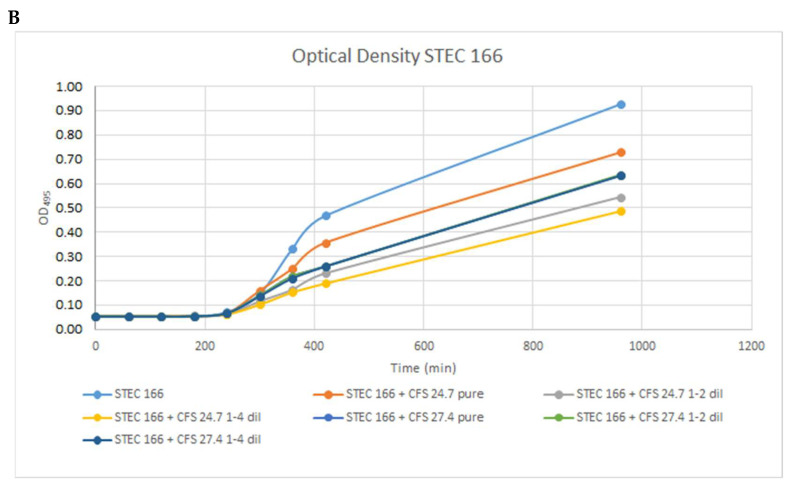
Results of the inhibitory activity of the colicins produced by the strains ColEc 24.7 and ColEc 27.4. (**A**) ColEc CFS inhibitory activity against STEC strain (log_10_ CFU/mL). (**B**) Optical density curves at 495nm and Log10 CFU/mL of STEC strain. Cultured in the presence of different SLC concentrations (pure, two-fold, and four-fold dilutions) of the antimicrobial substances of the selected strains.

Results of the inhibitory activity of the colicins produced ColEc 24.7 and 27.4 strains against STEC 166 are shown in Figure 2. These results showed a difference in the antimicrobial activity of the CFS analyzed. The CFS of ColEc 27.4 showed potential bactericidal activity against pathogenic strains (Figure 2A) when determining the OD, observed by the absence of growth in the early stages and the absence of viable cells when plate assays were performed. These results are indicative of a bactericidal effect. On the other hand, we documented a very slight decrease in the viability of pathogenic strains exposed to SFC of ColEc 24.7, although this effect was not maintained over time. The optical density and viable cells were lower and proportional to the dilution of CFS used (Figure 2B). These results show an antibacterial activity of ColEc 27.4 CFS on STEC strains. 

### 3.4. Differentiation Assay of Growth Inhibition by Phage or Colicin

In the six ColEc strains used, it was shown that the inhibition was due to the presence of bacteriocins and not of bacteriophages since confluent growth of the indicator strain was observed in all LB agar plates, with no lysis plaques indicating the presence of bacteriophages appearing in any of them.

### 3.5. Stability Assays

CFS from ColEc strains subjected to temperature, pH, and protease treatments showed a differential behavior. CFS from ColEc 24.7 was active after freezing and thawing at room temperature (25 °C), it also resisted a 10 min boiling at 100 °C but lost their inhibitory activity when incubated between 60 and 75 °C. When subjected to pH shifts, it only remains active at pH 7, while there was no activity at pH 4; 5; 9; 10. CFS from ColEc 27.4 showed inhibition subjected to freeze/thawing at room temperature, boiled for 10 min at 100 °C, and incubated between 60 and 75 °C. This strain of CFS also resisted pH variations maintaining its activity at pH 4; 5; 7; 9; 10. Both strains lost their inhibitory activity after Proteinase K incubation, allowing us to affirm the peptide nature of the antimicrobial substance in the CFS. 

### 3.6. Virulence Finder 

The sequenced ColEc strains (24.7 and 27.4) were negative for the Shiga toxin-associated genes (*stx11* and *stx2*) both by PCR in previous studies [31] and in the search through the Virulence Finder service. These genes are the most important virulence factors associated with STEC. 

### 3.7. Whole Genome Sequencing and Annotation: Bacteriocin Mining

The whole genome sequencing of ColEc 24.7 showed a guanine-cytosine (GC) percentage of 50.23%, with 717 contigs, while ColEc 27.4 presented a 50.56% GC in a total of 44 contigs. Annotation using PROKKA, after whole genome sequencing, confirmed the bacteriocins identified by PCR; additionally, we were able to detect the presence of Colicin, E1 in *E. coli* 24.7 and Colicin Ib in *E. coli* 27.4 that had not been identified by PCR.

**Figure 3:**
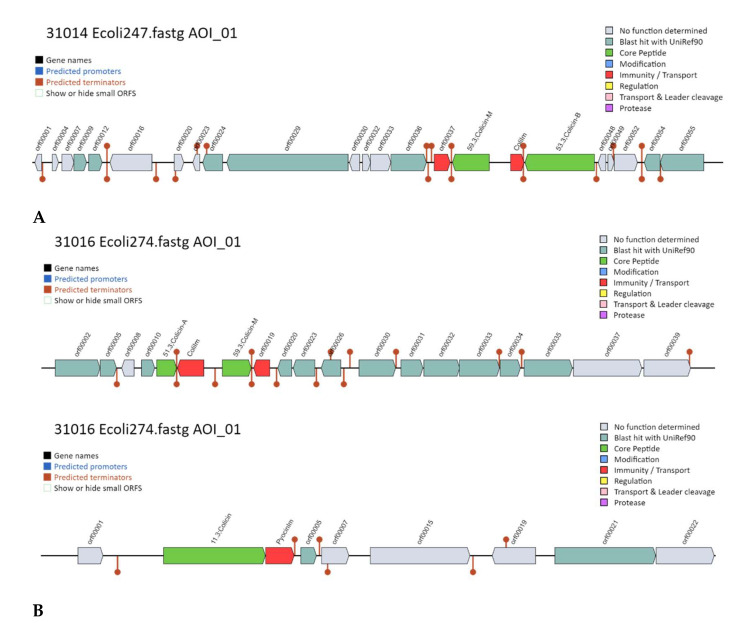
Bacteriocin Mining with BAGEL4. (**A**) Area of Interest (AOI) for ColEc 24.7 BAGEL4 showing Colicin M and Colicin B. (**B**) ColEc 27.4 1: AOI for Col Ib. 2: AOI for Col E7, Col E2 immunity gene, and Col E8 lysis gene AOI showing: Col B and Col M. Colicin core peptide gene (green) and immunity genes (red).

BAGEL4 analysis showed in ColEc 24.7 the presence of 2 AOI from a total of 205 that coded for bacteriocins or other ribosomally synthesized and post-translationally modified peptides (RiPPs) (Figure 3A). In one of these areas, we were able to identify the presence of Colicin M and Colicin B structural peptide genes as well as their respective immunity proteins.

ColEc 27.4 presented 4 AOI from a total of 88 areas analyzed (Figure 3B). AOI 01 presented the Col Ib gene; AOI 02 presented the Col E7 gene, Col E2 immunity gene, and Col E8 lysis protein; and AOI 04 had Col A, Col B, and their respective immunity genes (Figure 3B).

## 4. Discussion

Numerous research groups have identified bacteriocin-producing strains capable of inhibiting pathogens such as enterotoxigenic *Escherichia coli* (ETEC) and STEC [25,41,42]. The use of non-pathogenic commensal strains of *E. coli* is promising since they present high specificity by inhibiting only phylogenetically related pathogenic strains. *E. coli* Nissle 1917 stands out, marketed in some European countries under the name Mutaflor^®^ (©Pharma-Zentrale, Herdecke, Germany) whose inhibitory properties have been widely characterized, exhibiting microcin M/H47 production [43,44]. Administration of *E. coli* strains with antimicrobial activity available in commercial preparations has shown to outcompete and become dominant among the Enterobacteriaceae genus in the gut microbial communities of newborn and infant children [45]. The search for new strains and new molecules with comparable activity is a field of development [26,46,47]. Nevertheless, there has been criticism of the usage of Nissle 1917 as a probiotic due to several issues, such as the presence of a pathogenicity island called *pks*, which codifies and expresses a toxin called colibactin, a protein with a DNA alkylation activity that has been demonstrated to produce cross-linking damage on epithelial cell’s DNA [48]. This damage may lead to serious consequences such as colorectal cancer. A study led by Plequezuelos-Manzano in 2020 [49] was able to link the *pks* island to the mutational signature of colorectal cancer cells previously exposed to *E. coli.* In this regard, there have been studies focused on minimizing *E. coli* Nissle 1917 virulence factors using genome editing techniques such as CRISPR interference, this work made by Azam and Khan was able to knock down biofilm-related gene *csgD* and suppress virulence genes of *csgA*, *csgB*, *fimA*, *fimH*, *ompR*, *luxS*, and *bolA* related to *fimbriae*, two-component systems, *quorum* sensing and other functions on pathogenicity [50]. 

The ability to translocate through the intestinal epithelium to other tissues is a major concern on the safety of the probiotic strain. Pradhan and Weiss were able to prove, using human intestinal organoids, that *E. coli* Nissle was unable to cross the epithelial barrier and not affect the cells while other UPEC and EHEC bacteria did [51]. 

ColEc strains used were characterized as colicin producers [31] and grouped according to their genetic profiles (RAPD PCR). ColEc strains 4.8 and 24.7 share a genetic profile and harbor the same colicin and microcin genes. The profile of the other strains does not directly correlate to the presence of their colicin genes. Although they share a genetic profile, the serotypes observed differ between these strains. These differences may be due to a lack of discriminatory capacity of the RAPD technique.

The use of bioinformatic tools for sequence analysis is a robust and constantly growing field, the quality and quantity of sequence databases are an important factor to consider [52]. Sequence mining is a strategy to identify putative antimicrobial genes of interest in a specific bacterium. There are many platforms focused on these objectives, and we can highlight Bactibase [53], based on the Blast algorithm, which is a curated database for bacteriocins. AntiSMASH is another platform focused not only on bacteriocin but including other RiPPs. BAGEL4 is a database and mining suite with an advantageous user interface representing the AOI in a graphical layout displaying the genetic context of each putative bacteriocin. Some examples of the usage of these tools were the ones achieved by Cameron [54], who was able to identify multiple colicin and microcin genes (complete and truncated) in a sample obtained from soil, wastewater, and feces from feedlots in Alberta (Canada) combining sequence analysis on Bactibase and BAGEL4. Sabino [55] used BAGEL4 and AntiSMASH to detect lasso peptides produced by ruminal bacteria. In this work, we were able to detect previously unidentified bacteriocins (microcins and colicins) by combining PCR, genome sequencing, annotation, and mining for antimicrobial genes. This tool allowed us to confirm that the stx1 and stx2 genes were absent in the colicinogenic strain’s genome. This feature is of importance due to the severity of the HUS disease induced by the Shiga toxin. 

Colicins can present three mechanisms of destruction of target cells: (i) creation of voltage-gated channels in the inner membrane of the target bacterium, (ii) action of a nuclease in the cytoplasm (DNase, 16S rRNase, and tRNase), or (iii) inhibition of peptidoglycan synthesis. This study identified and characterized the colicins produced by ColEc strains demonstrating that they have inhibitory activity against STEC O157:H7 strains, being able to be used as an alternative tool in the control of that pathogenic strains. 

In this work, colicins and microcins have been detected with bacteriocin gene mining that was absent in PCR or genome annotation. These differences may be due to the quality of the assemblies obtained by the sequencing procedure. Genome annotation and mining tools are not without limitations due to the tedious and automated nature of these methods and the curation of genome databases compared to the mining tools. Several authors have approached this issue, such as Poptsova and Gogarten [56] and Kasaa [57].

This work showed that the ColEc strains under study could inhibit the bacterial growth of a high percentage of STEC O157:H7 strains obtained from clinical samples of patients with HC and HUS. Only a small percentage of the pathogenic strains showed resistance to the ColEc strains (Table 3). This percentage, although it is minor, is common to ColEc strains and deserves to be addressed in another study due to the dangerousness of the strains, and even subjected to sensitivity tests with other antimicrobial agents. These results indicate that colicinogenic strains and the inhibitory substances produced could be used to control STEC strains. The production of colicins in *E. coli* strains represents an important trait with respect to microbial survival and competition in the complex intestinal environment. A study by Micenková et al. [58] showed a new type of colicin, colicin Z, with a narrow inhibitory spectrum, being active only against enteroinvasive strains of *E. coli* (EIEC) and *Shigella*. ColE1 was produced from an *E. coli* K-12 strain containing the plasmid pColE1-K53 and effectively reduced *L. monocytogenes* populations in broth culture and on meat product surfaces [59]. Tahamtan et al. indicated that the use of colicin and biotherapy, rather than antibiotics, may be more effective for the control of E. coli K99 infection [60]. Therefore, the ability of colicinogenic strains to inhibit strains associated with important public health diseases such as HUS is remarkable. The antimicrobial substances present in the CFS of ColEc 27.4 maintained their activity against physical-chemical treatments of pH and temperature. This characteristic offers a potential application as antimicrobial agents on food to prevent or minimize the impact of bacterial contamination. The presence of virulence factors related to adherence and hemolysis is a serious concern about the potential application of a strain as a probiotic. However, this factor can be overcome by using antimicrobial substances such as colicins and microcins. These could be produced and purified en masse or by the cloning of the corresponding genes and factors necessary to achieve their expression and production in a safer microorganism.

Colicins and other bacteriocins are an alternative to antibiotics due to their comparatively narrow activity spectrum and relatively low frequency of resistance. Gillor et al. [61] reviewed potential applications of colicins and microcins to replace antibiotics in human or veterinary use or to apply them as food additives to prevent contamination with pathogens. However, to prevent the emergence of resistance, a combination of various colicins or microcins with distinct activities, different target receptors, or even molecular manipulation is a plausible strategy. Although combinations need to be evaluated, susceptible cells will have selective pressure on diverse points, and they will be, potentially, more constrained by tradeoffs and low fitness because of the metabolic ways involved in the resistance [62]. Our study shows that colicins are potent growth inhibitors of STEC O157:H7, being a potential alternative to reduce the presence of pathogens of public health relevance, and an alternative to use against multiresistant bacteria.

There are still pending issues arising from this study. It is interesting to study the resistance of STEC that did not display sensitivity against ColEc. A comparative study with other antimicrobial tools for the control of these pathogens could be carried out. The extraction and purification of colicins and microcins will be essential for their application in new pathogen control studies in biotechnological processes. And fundamentally, in vivo studies will be necessary to confirm its Generally Recognized As Safe character.

One of the most relevant aspects of colicins is the specificity and diversity of interactions that they undergo with various proteins during their action and during their production and release. The study of colicins, once the model for studies of bacterial toxins, has contributed significantly to progress in several fields. Colicins play some role in microbial communities. Researchers argue that they play a critical role in mediating microbial interactions [13]. The mystery not yet resolved involves knowing what that role is. Colicins can serve as anti-competitors, allowing a strain to invade an established microbial community. They can also play a defensive role and act to prohibit the invasion of other strains or species in an occupied niche or limit the advance of neighboring cells. It is likely that whatever roles bacteriocins play, these roles will change as components of the environment, both biotic and abiotic, change. 

There is a need to further investigate the importance of colicins and microcins with respect to the impact of interactions with prokaryotic and eukaryotic cells, as well as their putative use in food biotechnology and medicine. Considering the search for new antimicrobial and anticancer agents, Enterobacteriaceae, and especially colicins and microcins, should be considered valid options.

## Figures and Tables

**Table 1:** STEC O157:H7 strains used as indicator strains for the inhibitory activity of ColEc.

*n*	Code	Serotype	Virulence Genes	Origin	*n*	Code	Serotype	Virulence Genes	Origin
1	SHU-26	O157:H7	*stx2 eae*	HUS	41	BD 99	O157:H7	*stx2, eae, hlyA*	HC
2	E65-01	O157:H7	*stx2 eae*	HUS	42	BD 104	O157:H7	*stx2, eae, hlyA*	HC
3	E89-01	O157:H7	*stx2 eae*	HUS	43	BD 105	O157:H7	*stx2, eae, hlyA*	HC
4	E104-01	O157:H7	*stx2 eae*	HUS	44	BD 111	O157:H7	*stx2, eae, hlyA*	HC
5	E109-01	O157:H7	*stx2 eae*	HUS	45	9	O157:H7	*stx2, eae, hlyA*	HUS
6	E110-01	O157:H7	*stx2 eae*	HUS	46	HUS 58	O157:H7	*stx2, eae, hlyA*	HUS
7	E111-01	O157:H7	*stx2 eae*	HUS	47	72	O157:H7	*stx2, eae, hlyA*	HUS
8	E112-01	O157:H7	*stx2 eae*	WD	48	HUS 73	O157:H7	*stx2, eae, hlyA*	HUS
9	E113-01	O157:H7	*stx2 eae*	WD	49	76	O157:H7	*stx2, eae, hlyA*	HUS
10	E114-01	O157:H7	*stx2 eae*	WD	50	77	O157:H7	*stx2, eae, hlyA*	HUS
11	E115-01	O157:H7	*stx2 eae*	WD	51	INE 84-3	O157:H7	*stx2, eae, hlyA*	HUS
12	E116-01	O157:H7	*stx2 eae*	WD	52	123	O157:H7	*stx2, eae, hlyA*	HUS
13	E109-02	O157:H7	*stx2 eae*	WD	53	HUS 125	O157:H7	*stx2, eae, hlyA*	HUS
14	E92-02	O157:H7	*stx2 eae*	HUS	54	HUS 126	O157:H7	*stx2, eae, hlyA*	HUS
15	E44-02	O157:H7	*stx2 eae*	HUS	55	HUS 127	O157:H7	*stx2, eae, hlyA*	HUS
16	E42-02	O157:H7	*stx2 eae*	WD	56	144	O157:H7	*stx2, eae, hlyA*	HUS
17	E123-01	O157:H7	*stx2 eae*	HC	57	147	O157:H7	*stx2, eae, hlyA*	HUS
18	E117-01	O157:H7	*stx2 eae*	WD	58	148	O157:H7	*stx2, eae, hlyA*	HUS
19	E118-01	O157:H7	*stx2 eae*	WD	59	HUS 156	O157:H7	*stx2, eae, hlyA*	HUS
20	E309-02	O157:H7	*stx2 eae*	HUS	60	HUS 161	O157:H7	*stx2, eae, hlyA*	HUS
21	E192-02	O157:H7	*stx2 eae*	WD	61	HUS 179	O157:H7	*stx2, eae, hlyA*	HUS
22	E165-02	O157:H7	*stx2 eae*	WD	62	HUS 196	O157:H7	*vt1, stx2, eae, hlyA*	HUS
23	E161-02	O157:H7	*stx2 eae*	WD	63	INE 198-4	O157:H7	*stx2, eae, hlyA*	HUS
24	E121-02	O157:H7	*stx2 eae*	WD	64	226	O157:H7	*stx2, eae, hlyA*	HUS
25	E111-02	O157:H7	*stx2 eae*	WD	65	230	O157:H7	*stx2, eae, hlyA*	HUS
26	E110-02	O157:H7	*stx2 eae*	WD	66	238	O157:H7	*stx2, eae, hlyA*	HUS
27	E310-03	O157:H7	*stx2 eae*	WD	67	HUS 239	O157:H7	*stx2, eae, hlyA*	HUS
28	E211-03	O157:H7	*stx2 eae*	HC	68	272	O157:H7	*stx2, eae, hlyA*	HUS
29	E205-03	O157:H7	*stx2 eae*	HC	69	291	O157:H7	*stx2, eae, hlyA*	HUS
30	E121-03	O157:H7	*stx2 eae*	HC	70	HUS 303	O157:H7	*stx2, eae, hlyA*	HUS
31	E111-03	O157:H7	*stx2 eae*	HC	71	307	O157:H7	*stx2, eae, hlyA*	HUS
32	E92-03	O157:H7	*stx2 eae*	HUS	72	HUS 312	O157:H7	*stx2, eae, hlyA*	HUS
33	E16-03	O157:H7	*stx2 eae*	HC	73	HUS 316	O157:H7	*stx2, eae, hlyA*	HUS
34	E14-03	O157:H7	*stx2 eae*	HC	74	HUS 331	O157:H7	*stx2, eae, hlyA*	HUS
35	GB	O157:H7	*stx2 eae*	HC	75	HUS 349	O157:H7	*stx2, eae, hlyA*	HUS
36	E30-00	O157:H7	*stx2 eae*	HUS	76	HUS 352	O157:H7	*stx2, eae, hlyA*	HUS
37	E3-99	O157:H7	*stx2 eae*	HUS	77	HUS 357	O157:H7	*stx2, eae, hlyA*	HUS
38	E93-03	O157:H7	*stx2 eae*	HC	78	358	O157:H7	*stx2, eae, hlyA*	HUS
39	BD 8	O157:H7	*stx2, eae, hlyA*	HC	79	HUS 360	O157:H7	*stx2, eae, hlyA*	HUS
40	BD 14	O157:H7	*stx2, eae, hlyA*	HC	80	HUS 361-4	O157:H7	*stx2, eae, hlyA*	HUS

WD: Watery Diarrhea; HC: Hemorrhagic Colitis; HUS: Hemolytic Uremic Syndrome.

**Table 2:** Colicin primers used in PCR for colicin genes identification.

Colicin	Primer	Sequence	Amplicon Size (Bp)
B	colicinB-F	AAGAAAATGACGAGAAGACG	493
	colicinB-R	GAAAGACCAAAGGCTATAAGG	
E1	colicinE1-F	TGTGGCATCGGGCGAGAATA	650
	colicinE1-R	CTGCTTCCTGAAAAGCCTTTT	
E7	ColE7-F	GCATTCTGCCATCTGAAAT	431
	ColE7-R	CTTCTGCCCACTTTCTTTCG	
Ia	ColIa-F	GCATGCAAATGACGCTCTTA	473
	ColIa-R	GAGGACGCCAGTTCTCTGTC	
K	ColK-F	CAGAGGTCGCTGAACATGAA	469
	ColK-R	TCCGCTAAATCCTGAGCAAT	
M	ColM-F	GCTTACCACTTCGCAAAACC	429
	ColM-R	GAGCGACTCTCCGATAATGC	
S4	ColS4-F	TATATGGCCCAACTGCTGGT	456
	ColS4-R	CGTAAGGACGGACACCTGTT	
B17	microcin B17-F	TCACGCCAGTCTCCATTAGGTGTTGGCATT	135
	microcin B17-R	TTCCGCCGCTGCCACCGTTTCCACCACTAC	
H47	microcin H47-F	CACTTTCATCCCTTCGGATTG	227
	microcin H47-R	AGCTGAAGTCGCTGGCGCACCTCC	
J25	microcin J25-F	TCAGCCATAGAAAGATATAGGTGTACCAAT	175
	microcin J25-R	TGATTAAGCATTTTCATTTTAATAAAGTGT	
V	microcin V-F	CACACACAAAACGGGAGCTGTT	680
	microcin V-R	CTTCCCGCAGCATAGTTCCAT	

**Table 3:** Inhibition test of ColEc strains on STEC strains isolated in clinical cases of HUS, HC, and WD and the STEC 166 strain isolated from grazing cattle (*n* = 81) and results de colicins and microcins production.

Colicigenic *E. coli* Strains		STEC Strains Inhibited	Colicin	Microcin
ID Strain	Serogroup	% (Positive Reading)		
4.8	ONT	90.1 (73)	B	M, H47
8.2	O77	90.1 (73)	Ia	C7
13.7	O174	95.1 (77)	Ia, E7, B	C7, J25
24.7	ONT	95.1 (77)	B	M, H47
27.4	O26	92.6 (75)	Ia, E7, B	C7, J25
27.12	O141	92.6 (75)	-	-

## Data Availability

Data is contained within the article.
